# Comprehensive Review: Machine and Deep Learning in Brain Stroke Diagnosis

**DOI:** 10.3390/s24134355

**Published:** 2024-07-04

**Authors:** João N. D. Fernandes, Vitor E. M. Cardoso, Alberto Comesaña-Campos, Alberto Pinheira

**Affiliations:** 1 INESC TEC, 4200-465 Porto, Portugal; 2 Collaborative Laboratory for the Future Built Environment (BUILT CoLAB), Rua Do Campo Alegre, 760, 4150-003 Porto, Portugal; vitor.cardoso@builtcolab.pt; 3 Faculty of Engineering, University of Porto, 4200-465 Porto, Portugal; 4 Department of Computer Engineering, Superior Institute of Engineering of Porto, 4249-015 Porto, Portugal; gpa@isep.ipp.pt; 5 Department of Design in Engineering, University of Vigo, 36312 Vigo, Spain; acomesana@uvigo.gal; 6 Design, Expert Systems and Artificial Intelligent Solutions Group (DESAINS), Galicia Sur Health Research Institute (IIS Galicia Sur), SERGAS-UVIGO, 36312 Vigo, Spain; 7 Center for Health Technologies and Information Systems Research—CINTESIS@RISE, Faculty of Medicine, University of Porto, 4200-450 Porto, Portugal

**Keywords:** brain stroke, deep learning, machine learning, classification, segmentation, object detection

## Abstract

Brain stroke, or a cerebrovascular accident, is a devastating medical condition that disrupts the blood supply to the brain, depriving it of oxygen and nutrients. Each year, according to the World Health Organization, 15 million people worldwide experience a stroke. This results in approximately 5 million deaths and another 5 million individuals suffering permanent disabilities. The complex interplay of various risk factors highlights the urgent need for sophisticated analytical methods to more accurately predict stroke risks and manage their outcomes. Machine learning and deep learning technologies offer promising solutions by analyzing extensive datasets including patient demographics, health records, and lifestyle choices to uncover patterns and predictors not easily discernible by humans. These technologies enable advanced data processing, analysis, and fusion techniques for a comprehensive health assessment. We conducted a comprehensive review of 25 review papers published between 2020 and 2024 on machine learning and deep learning applications in brain stroke diagnosis, focusing on classification, segmentation, and object detection. Furthermore, all these reviews explore the performance evaluation and validation of advanced sensor systems in these areas, enhancing predictive health monitoring and personalized care recommendations. Moreover, we also provide a collection of the most relevant datasets used in brain stroke analysis. The selection of the papers was conducted according to PRISMA guidelines. Furthermore, this review critically examines each domain, identifies current challenges, and proposes future research directions, emphasizing the potential of AI methods in transforming health monitoring and patient care.

## 1. Introduction

Brain stroke, also known as a cerebrovascular accident, is a critical medical condition that occurs when the blood supply to part of the brain is interrupted or reduced, preventing brain tissue from receiving oxygen and nutrients. This condition can lead to devastating consequences, including paralysis, difficulties in speech, and sometimes memory loss, among others [1]. Brain strokes can be divided primarily into two types: ischemic stroke, which is caused by blockages (such as blood clots), and hemorrhagic stroke, caused by bleeding in or around the brain [2]. According to the World Health Organization [3], 15 million people worldwide suffer a stroke per year. Among those, 5 million die, and another 5 million end up being permanently disabled. While brain stroke commonly occurs in people over 40 years old, it can also strike younger individuals and is the main cause of high blood pressure in those under 40. In children, particularly those with sickle cell disease, stroke occurs in about 8% of cases [4,5,6]. Older adults are particularly susceptible to brain strokes due to physiological changes that accompany aging, such as the stiffening of blood vessels and the increased prevalence of age-related diseases such as atrial fibrillation, hypertension, and diabetes. These factors significantly elevate the risk of brain stroke. Furthermore, recovery in older patients can be complicated by pre-existing health conditions, where the impact of brain stroke can be more severe, leading to greater functional decline and a need for long-term care. Environmental and socioeconomic factors, such as pollution exposure and limited healthcare access, further compound these risks. Preventative strategies for all ages, including lifestyle modifications and the management of underlying conditions, are crucial to mitigating the risk of brain stroke [7].

The complexity and interplay of brain stroke risk factors underscore the need for advanced data processing, analysis, and fusion techniques for a comprehensive health assessment. Machine learning (ML) and deep learning (DL) offer promising solutions in this area. By analyzing vast datasets on patient demographics, health records, lifestyle choices, and genetic information, ML and DL algorithms can uncover patterns and predictors of brain stroke risk that are not immediately apparent to humans. This capability is crucial for the development of predictive health monitoring systems and personalized care recommendations, optimizing healthcare resources and improving patient outcomes.

Moreover, the performance evaluation and validation of advanced sensor systems in brain stroke diagnosis through image recognition algorithms are essential. These technologies quickly and accurately interpret magnetic resonance imaging (MRI) and Computed Tomography (CT) scans, potentially speeding up the decision-making process for treatments such as clot-busting medications or surgical interventions [8,9].

As we delve deeper into the potential of ML and DL in the context of brain stroke management, it becomes clear that integrating technology with traditional healthcare approaches can lead to early detection and therefore to possible mitigation actions [10]. This paper aims to provide a comprehensive review of review papers within the field of ML and DL in the clinical analysis of brain strokes. Our focus is primarily on three interconnected ML and DL approaches that are crucial for diagnosis: classification, segmentation, and object detection problems. Classification algorithms identify whether a brain stroke has occurred and classify its type; segmentation techniques localize the affected brain areas, and object detection algorithms highlight abnormalities in brain scans. These methods collectively improve diagnostic accuracy and are vital for developing targeted therapeutic strategies. The legitimacy and relevance of focusing on these three areas are well-supported by the existing literature, for example, the review study of Yedavalli et al. [11]. Moreover, to the best knowledge of the authors, this is the first comprehensive review that collects and discusses the most pertinent literature reviews across these fields within a single paper.

Following this introduction, Section 2 will provide a theoretical overview of the three fields in ML and DL. Section 3 will describe the search process used to collect the most relevant literature, including a detailed analysis of common applications of ML and DL in brain stroke detection, and a review of crucial papers on classification problems in stroke analysis. Based on this search process, several research questions are formulated to be addressed by the end of the paper. In Section 4, application fields will be introduced and discussed, summarizing key findings and methodologies from major review papers in a tabular format. Additionally, Section 4 will present the most relevant datasets in brain stroke management. Section 5 will outline the challenges and suggest future directions to assist researchers in this area, based on our analysis of the review papers, aiming to enhance the integration of ML and DL technologies within traditional healthcare frameworks for better stroke management. In Section 6, we will address the formulated research questions based on our conclusions and inferences from the review papers. Finally, in Section 7, we will draw our conclusions.

## 2. Theorethical Overview

Before delving into the comprehensive review of the most pertinent literature in each field of ML and DL related to brain stroke, this section will first provide a theoretical overview of the primary methodologies employed: classification, segmentation, and object detection. The aim is to outline the fundamental formulations and applications of these techniques. This overview will not only set the stage for the detailed review to follow but also serve as a guide for new researchers in the fields of ML and DL, helping them to understand the foundational concepts and direct their learning efforts more effectively.

**Figure 1:**
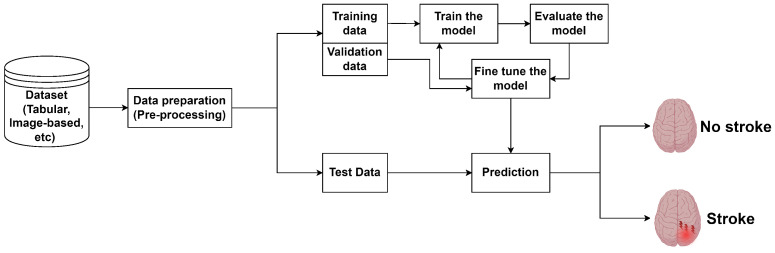
Example of a classification problem.

Classification is aimed at categorizing data into distinct groups or classes. This is achieved by employing algorithms that analyze training data, which are pre-labeled with the correct output, to discern patterns that can be applied to new, unseen data. The mathematical formulation of such algorithms often holds on probability theory, logical, and geometrical problems. Such algorithms are typically handled through supervised learning, where a model is trained on a dataset that includes both the features (inputs) and the labels (outputs). As models are built and refined, their performance must be quantitatively evaluated. This involves metrics like accuracy, precision, recall, and the F1 score, all of which provide insights into the model’s efficacy and areas for potential improvement. The most common algorithms applied encompass Logistic Regression (LR), Naïve Bayes (NB), Decision Trees (DTs), Support Vector Machines (SVMs), and ensemble algorithms, which typically include Random Forests (RFs) and Gradient Boosting (GB) algorithms. For more detailed reading concerning these algorithms and their formulations, readers are directed to [12,13,14,15,16,17,18,19,20,21]. Concerning classification problems in DL, the basic computational unit of a deep neural network is the neuron. Each neuron receives inputs, applies a set of weights to these inputs, adds a bias, and passes the result through a non-linear activation function. For a single neuron, this process can be described mathematically, as its output is

(1) $$a=f(w^{T}x+b)$$

where *f* commonly represents a non-linear activation function, such as ReLU, which introduces the ability to model non-linear relationships within the data. DL networks learn through an iterative process called backpropagation, where the network adjusts its weights to minimize the error in its predictions. The error is quantified using a cost function, such as cross-entropy loss in classification tasks. For a deeper understanding of DL networks, the readers are directed to [12,13,14,15,16,17,18,19,20,21]. Figure 1 shows a typical process for a classification problem.

**Figure 2:**
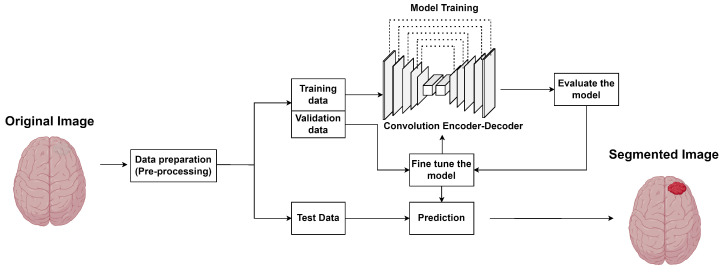
Example of a segmentation problem.

Segmentation is a critical task in deep learning where the goal is to partition an image into segments, or pixels, with each segment corresponding to different objects or regions of interest. In the context of deep learning, segmentation models are designed to understand and delineate the boundaries of objects within images, making it a fundamental tool for image analysis and interpretation. The most common segmentation models are Convolutional Neural Networks (CNNs). They are specialized deep-learning architectures that learn spatial hierarchies of features from images. For segmentation tasks, CNNs can be trained to classify each pixel in an image, thus segmenting the image into meaningful regions. Figure 2 shows a typical segmentation problem.

**Figure 3:**
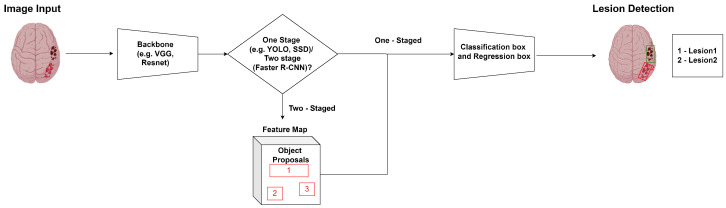
Example of an object detection problem.

Object detection, a critical task in computer vision, extends beyond classification and segmentation by not only identifying the objects present in an image but also pinpointing their location with bounding boxes. This capability enables a deeper understanding of images by providing not just the “what” but also the “where” of objects, making it essential for applications like surveillance, image medical synthesis, autonomous driving, and structural damage recognition, among others. Object detection algorithms can be broadly categorized into one-stage and two-stage approaches. One-stage algorithms, such as You Only Look Once (YOLO) and single-shot detectors (SSDs) perform detection and classification in a single pass, offering faster processing times. Two-stage algorithms, such as Region Convolutional Neural Networks (Faster R-CNN), first generate region proposals and then classify these regions, typically achieving higher accuracy. Figure 3 shows a typical object detection problem.

It is noteworthy to mention that several models can be applied to solve each of these mentioned tasks. Moreover, Table 1 summarizes the most relevant models, with the corresponding paper reference so readers can be directed. Moreover, the most common metrics as well as the losses employed in each task are also described in the table.

## 3. Search Strategy

**Figure 4:**
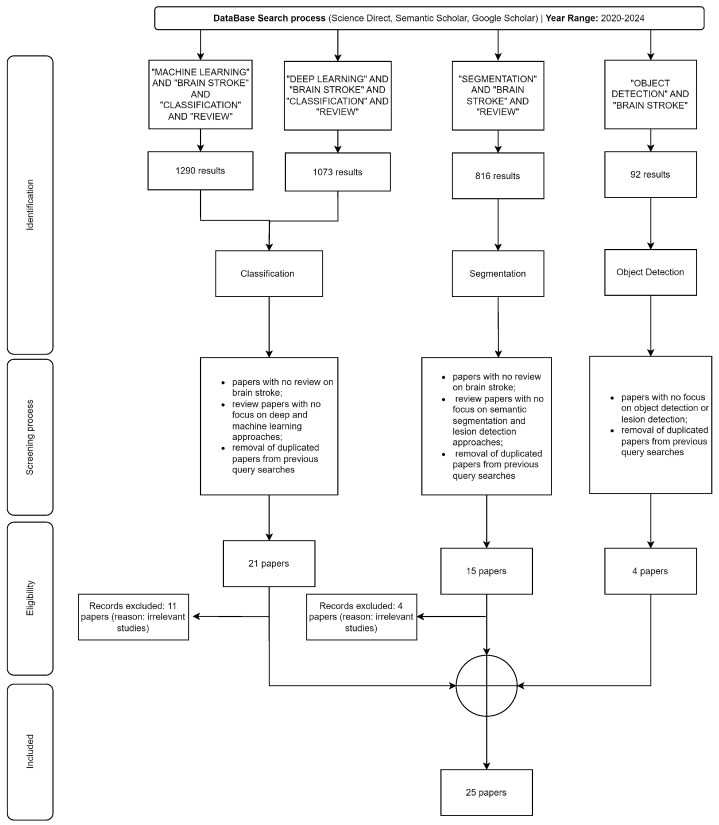
Search strategy.

The search strategy was designed to explore the most pertinent aspects of brain stroke analysis using ML and DL techniques. The review encompassed the literature from the ScienceDirect database, featuring papers published by Elsevier. Additionally, the Semantic Scholar database was consulted, which included articles from IEEE, PubMed, Taylor and Francis, MDPI, PLOS ONE, Springer, Hindawi, Frontiers, and SAGE journals. The selection of papers was based not only on keyword searches but also on their relevance, as indicated by the number of citations and the Frequency of CitedWorks Indicator (FCWI). Figure 4 shows the selection of the papers for the different fields based on the keyword search. It is important to note that PRISMA [38] guidelines were used to outline the retrieval process.

Following the screening strategy, 10 papers discuss issues related to classification in ML or DL. Given its fundamental nature, it is unsurprising that many review papers focus on classification tasks related to brain strokes. This focus suggests a strong, established interest in using ML and DL, to classify types of brain strokes or predict outcomes based on imaging, symptoms, or other clinical data. Additionally, 11 review papers address segmentation issues. In DL, particularly in medical imaging, segmentation involves dividing an image into segments to simplify its representation or to make it more meaningful and easier to analyze. It is commonly used to identify regions of interest, such as stroke lesions in brain scans. The number of papers on segmentation roughly equals those on classification, highlighting its significance in precisely localizing affected areas in medical images. Moreover, four papers explore object detection issues. Object detection, a distinct field from segmentation, aims to localize regions of interest by drawing bounding boxes around objects rather than segmenting them. This approach integrates elements of both classification and segmentation to locate and classify individual objects within an image. Object detection is a more specialized task than the broader applications of classification and segmentation, which may explain the fewer papers on this topic. This disparity could indicate that object detection is either a newer or less explored area in the context of brain stroke within the Artificial Intelligence (AI) community. The higher numbers of review papers in classification and segmentation likely reflect their direct applicability and urgency in clinical settings for diagnosis and treatment planning. Moreover, areas like object detection present fewer research papers, compared with classification and segmentation, due to their difficulty in application when compared with classification and segmentation. This strategic search, therefore, leads to formulating Research Questions (RQs) of paramount importance in the field of ML and DL concerning brain stroke, such as the following:

- RQ1: What are the cases where machine learning and deep learning are more appropriate for building a robust and accurate model for classification problems?
- RQ2: What are the challenges and limitations of current AI segmentation techniques in analyzing complex brain imaging data?
- RQ3: Why object detection studies are less studied when compared to segmentation?
- RQ4: What are the most prominent challenges and future directions in machine learning and deep learning considering stroke diagnosis?

## 4. Results on the Papers Found for Each Field in Brain Stroke Diagnosis

As stated in Section 3, the discussion of the papers will be conducted per field of application. Therefore, this section is subdivided into subsections, each representing the field of application. Furthermore, a final subsection will is also included to report the most relevant datasets found in the field of brain stroke analysis.

### 4.1. Classification

In the field of classification problems for brain stroke detection research, the integration of ML and DL has marked a transformative phase, bridging the gap between rapid diagnosis and effective treatment strategies. Sirsat et al. [1] provides a comprehensive review of studies, systematically categorizing them into these key areas, thereby offering invaluable guidance for researchers diving into specific aspects of stroke management using ML. This organized approach highlights the diverse applications of ML techniques in improving patient care and outcomes. Simultaneously, Wang et al. [39] focuses on ML in stroke imaging applications, highlighting the scarcity of comprehensive reviews that integrate structured data to predict stroke outcomes. They specifically note the predominant use of algorithms such as SVMs, RFs, DTs, and Artificial Neural Networks (ANNs) for mortality prediction, offering detailed insights through tabulated study characteristics.

Gagana and Padma [40] and Mushtaq and Saini [41] explore the efficacy of ML models in risk prediction, employing a mix of clinical, demographic, and imaging data to refine the accuracy of these models. The integration of such data not only improves the prediction outcomes but also enhances the practical utility of ML in clinical settings. Bashir et al. [42] extend this discussion to brain stroke detection, analyzing the performance and generalization capabilities of ML models across different frameworks.

Cui et al. [43] discuss the application of DL in managing acute ischemic stroke, detailing how DL models facilitate rapid and accurate assessments crucial for effective treatment planning. They particularly highlight the advancements in early diagnosis and functional outcome prediction. The work of Tan et al. [44] emphasizes the diagnostic superiority of CNNs in analyzing imaging data, which has significantly transformed ischemic stroke prediction.

In a comparative analysis, Bajaj et al. [45] and Daidone et al. [46] discuss the roles of various ML and DL techniques in stroke detection and the potential of these technologies in paving the way for precision medicine. They note the specific effectiveness of CNNs due to their capability to autonomously learn from complex imaging data.

Adding to the spectrum of reviewed studies, Ruksakulpiwat et al. [47] evaluate ML-based classification systems for stratifying stroke patients. They report the use of multiple ML algorithms, with SVM, RF, DT, and GB being predominant. Their findings highlight the importance of age and gender as frequent features in classification models, while also pointing out the variable usage of other critical data like glucose levels and hypertension status. This review emphasizes that no single algorithm universally outperforms others across all stroke classification tasks, underscoring the necessity to select algorithms based on specific data characteristics and clinical contexts.

Table 2, Table 3 and Table 4 show a summary of the aforementioned studies, highlighting the number of found review papers, the range of work found, key points of the paper, strengths, limitations, and challenges/opportunities.

### 4.2. Segmentation

Segmentation in medical imaging analysis represents a very important field in DL modeling since it provides valuable and critical information for tasks such as lesion detection, which is important for further clinical diagnosis. These technologies particularly leverage MRI and CT scans to provide precise, automated lesion segmentation, which is crucial for effective treatment planning.

A collection of reviews emphasized the utility of MRI for brain stroke lesion segmentation. Saad et al. [48], Isa et al. [49], and Subudhi et al. [50] discuss a range of manual to automated segmentation methods, including advanced machine learning models. These studies highlight the precision of MRI in detailing soft tissue contrast, making it highly effective for early stroke detection and characterization. The comparative benefits of MRI versus CT are critically analyzed in the literature. While MRI is favored for its detailed tissue contrast, the rapid imaging capabilities of CT scans make them indispensable in emergency settings, as discussed in Karthik et al. [51]. Nonetheless, the work of Inamdar et al. [52] further explores computer-aided systems in acute stroke diagnosis, demonstrating how computational models enhance diagnostic workflows.

DL models, particularly U-Net architectures, have revolutionized medical image segmentation. Liu et al. [53] explores various U-Net adaptations that enhance feature extraction and lesion delineation. Abbasi et al. [54] and Malik et al. [55] both highlight how CNNs and their variants have been instrumental in segmenting ischemic and hemorrhagic stroke lesions, integrating attention mechanisms and multi-dimensional networks to manage the complexities of neuroimaging data. [56] extends this discussion to brain tumors, assessing how deep learning outperforms traditional methods in tumor identification and classification.

Emerging trends such as the application of AI tools in chronic conditions are detailed in [57], which evaluates the effectiveness of AI in chronic stroke analysis. Thiyagarajan and Murugan [58] and Saad et al. [48] both stress the need for robust, scalable models capable of integrating multimodal data to improve diagnostic accuracy and operational efficiency. Table 5, Table 6 and Table 7 show a thorough summary of the main objectives of each review paper as well as strengths and limitations and some challenges that they propose.

### 4.3. Object Detection

Object detection is crucially important in the analysis of brain strokes, leveraging advanced imaging technologies to improve diagnostic accuracy and treatment efficacy. This task involves the identification and classification of various structures within the brain using algorithms that interpret complex imaging data. Concerning the context of brain stroke, object detection helps in the quick detection of areas of the brain affected by strokes (clots or hemorrhages), thus facilitating timely interventions. Since object detection enables detailed visualizations of the impact of a stroke, it becomes a valuable tool for supporting critical decisions regarding the most appropriate patient recovery strategies. It is noteworthy to mention that object detection is highly connected to segmentation in the field of medical imaging. While object detection focuses on the area by providing an output such as bounding boxes around the lesion found, segmentation goes further by dividing the image into segments and isolating these detected objects with precise boundaries. While conducting this comprehensive survey, the amount of work found directly with the keyword search of object detection was very scarce when compared to segmentation.

As stated in Section 3, the field of object detection does not offer as detailed and widely accepted state-of-the-art methodologies for brain stroke analysis compared to segmentation and classification. This is due to the relatively recent development of the methodologies employed in object detection and the complexity of extracting relevant features. While object detection is valuable, it often focuses on identifying discrete objects or anomalies within an image. In the context of brain strokes, objects such as blood clots might not be as discretely definable as in other applications, such as identifying tumors, making segmentation a more practical choice for analysis. Another reason is related to data availability and quality. High-quality annotated datasets are more common for segmentation than for object detection, which accelerates the improvement of segmentation techniques, therefore making a more viable tool for current brain stroke diagnosis methods. Object detection requires more precision in annotated data (i.e., location of boundaries), which are less commonly compiled in stroke-related datasets. Additionally, the clinical relevance of segmentation is significant. Segmentation directly aids in quantifying affected brain areas and visualizing damage, whereas object detection might not provide complementary insights, such as identifying smaller or emerging pathological features, which are crucial for immediate clinical decision making. Moreover, segmentation algorithms are easier to integrate into clinical workflows, providing clear and immediate benefits to medical professionals, such as radiologists or neurologists, who are responsible for assessing the extent of brain damage.

Therefore, due to the smaller body of literature, it is reasonable to analyze papers that focus more on the implementation of brain stroke detection and extrapolate findings from other review papers that do not focus specifically on brain stroke detection. The following Table 8, Table 9 and Table 10 summarize the aforementioned studies, highlighting key points, strengths, limitations, and challenges/opportunities, respectively, in brain stroke detection.

### 4.4. Relevant Datasets for Stroke Diagnosis

In building upon our examination of classification, segmentation, and object detection, it becomes evident that the efficiency of these techniques relies significantly on the quality and appropriateness of the datasets. In the domain of brain stroke diagnosis specifically, customized datasets are not just beneficial but essential for training robust models. These datasets must accurately reflect the heterogeneous nature of stroke symptoms and patient demographics to ensure that models are both precise and generalizable across real-world conditions. This section aims to present the datasets that are most relevant for stroke diagnosis, detailing their composition, source, and the unique challenges that they help address in the context of applying advanced diagnostic algorithms. A summary of the datasets can be seen in Table 11.

Among these, the Stroke Prediction Dataset is essential for developing tabular predictive models focused on risk assessment and early warning signs of stroke. The Brain MRI Segmentation and ISLES datasets are critical image datasets for training algorithms to identify and segment brain structures affected by strokes. The Cerebral Vasoregulation in Elderly with Stroke dataset provides valuable insights into cerebral blood flow regulation post stroke, useful for both tabular analysis and image-based modeling.

Additionally, the National Institutes of Health Stroke Scale (NIHSS) Annotations for the MIMIC-III dataset offer detailed annotations within a comprehensive tabular dataset, facilitating nuanced models that predict stroke severity and outcomes. The China National Stroke Registry (CPSS) combines clinical information and outcomes of stroke patients in China across both tabular and image data formats. For comparative analyses in stroke-related brain damage, the Brain Tumor Segmentation (BraTS2020) dataset, though primarily used for brain tumor studies, also provides relevant imaging data.

The Anatomical Tracings of Lesions After Stroke (ATLAS) dataset is designed for detailed studies of post-stroke lesions, supporting advanced segmentation tasks. Publicly accessible datasets like the Healthcare Dataset Stroke Data and the CDC Diabetes Health Indicators offer tabular data widely used in predictive modeling for stroke diagnosis and identifying stroke risk correlations. The Framingham Heart Study dataset and the Health and Retirement Study (HRS) dataset provide longitudinal data that has informed numerous models over decades, particularly in cardiovascular and cerebrovascular diseases. Lastly, the MIMIC dataset provides a rich source of both tabular and image data from intensive care units, including detailed stroke patient records.

While the datasets discussed previously are crucial for enhancing stroke diagnosis through advanced computational models, it is equally important to consider broader innovations in medical imaging that can further inform and improve these efforts. The methodologies and findings from studies such as Tian et al. [63], Wenxuan et al. [64], Zhang et al. [65], Wang et al. [66], Naval Marimont and Tarroni [67], Li et al. [68], and Tian et al. [69] highlight a spectrum of approaches that tackle various challenges in medical imaging. These studies not only push the boundaries of what is technologically possible but also provide valuable insights that could be adapted for more effective stroke diagnosis and treatment planning. For instance, the precision in localization and segmentation demonstrated in tumor studies could enhance the accuracy of identifying stroke-affected areas in the brain. Similarly, techniques developed for anomaly detection in unsupervised settings might be applied to identify unusual patterns in stroke imaging that precede visible symptoms, thereby enabling earlier intervention. By integrating knowledge from these diverse yet related areas of medical imaging, researchers and clinicians can develop more nuanced and powerful tools for diagnosing and treating strokes. This holistic approach ensures that advancements in one area can benefit broader medical applications, fostering innovation and improving outcomes across various domains of healthcare.

**Table 11:** Relevant datasets for stroke diagnosis.

Dataset	Reference	Type	Access
Stroke prediction dataset	[70]	tabular	Open
Brain MRI segmentation	[71]	images	Open
ISLES	[72]	images	Restricted
Cerebral Vasoregulation in Elderly with Stroke	[73]	tabular	Open
NIHSS Annotations for the MIMIC-III Database	[74]	tabular	Restricted
CPSS	[75]	tabular	Restricted
BraTS2020	[76]	images	Open
ATLAS	[77]	images	Open
Healthcare Dataset Stroke Data	[78]	Tabular	Open
CDC Diabetes Health Indicators	[79]	Tabular	Open
Framingham heart study dataset	[80]	Tabular	Open
MIMIC	[81]	Tabular	Open
HRS Dataset	[82]	Tabular	Open

## 5. Open Challenges and Future Directions

From the reviewed papers, we concluded that while ML and DL continue to advance brain stroke diagnosis through improved classification, segmentation, and object detection, several significant challenges persist. These challenges not only highlight the gaps in current methodologies but also outline potential areas for future research. Addressing these issues will be crucial for enhancing the diagnostic accuracy, reliability, and clinical applicability of ML and DL models. In this section, we identify and discuss key challenges and propose future research directions that, based on our analysis of the review papers, could lead to more robust, interpretable, and effective stroke diagnosis tools.

### 5.1. Need for Explainability and Interpretability

Explainable AI (xAI) seeks to address one of the significant challenges in deep learning and AI, the black-box nature of many AI models. As AI systems, particularly deep neural networks, become increasingly complex, understanding the decision-making process behind their predictions becomes both challenging and essential. Regulatory authorities have established policies that enforce greater accountability in algorithmic decision making [83]. These guidelines aim to ensure transparency and fairness in the use of algorithms across various sectors, particularly in the healthcare sector. Concerning the medical field, a great amount of the literature can already be found aiming to propose explainable algorithms to support the decision-making process [84,85,86,87,88,89,90,91,92]. Concerning the field of stroke diagnosis, there has been some work published. Gurmessa and Jimma [93] reviewed several works in the field of stroke analysis. Most of the works found by the authors point to Post Hoc approaches, i.e., additional algorithms are used to interpret black-box models. However, it is noteworthy to point out that other types of approaches could be used in this field. For example, intrinsically explainable methods are indeed more challenging to develop; however, it is possible to combine a single model with a hybrid approach with a transparent and black-box model. Although some performance could be lost, it would be gain in interpretability. One famous example of such an approach is the ProtoPNet [94]. Another issue that remains a challenge in explainable AI involves the adopted metrics. Rosenfeld [95] claimed that some studies wrongly assume that low explanations should be accepted for some tasks. However, the same author proposes some metrics to quantify the explainability. However, those metrics remain a challenge due to the vast amount of different domains across the literature.

### 5.2. Need for Generative Models

Generative AI encompasses models and techniques that focus on generating new data samples consistent with the distribution of a given dataset. Unlike discriminative models that predict a label or outcome, generative models can create new data instances, such as images, and tabular in the training data. This ability opens up the possibility of enriching the datasets and improving the performances of the models. At the moment, the most famous generative approaches found in the literature are the Generative Adversarial Networks (GANs) [96], Variational Autoencoders (VAEs) [97], and diffusion models [98]. In the field of medical analysis, some discussions have been presented in the literature, wherein it is noteworthy to mention Ali et al. [99], Laino et al. [100], Dayarathna et al. [101], Kazerouni et al. [102], Ferreira et al. [103]. These works have been provided through comprehensive reviews of medical image synthesis, highlighting their applications in generating images with high fidelity. Concerning the field of stroke diagnosis, a comprehensive review was conducted by Gong et al. [104] where the authors pointed out a work conducted by Wang et al. [105], where the Consistent Perception Generative Adversarial Network (CPGAN) was introduced to enhance the effect of brain stroke lesion prediction for unlabeled data. Although generative models present valuable tools for image generation, some challenges remain, especially in domain adaptation and domain generalization. The literature review claims that there is still a lot of space for improvement in the performance of the models when faced with a new domain unseen. Another interesting challenge to address is the usage of diffusion approaches for tabular data. So far, there are several works on image synthesis. However, many datasets on stroke diagnosis are tabular and most of the time, the number of samples is very low and highly imbalanced. Adopting generative approaches for tabular data could serve as an interesting study, as there are very few approaches on tabular data, and to the knowledge of the authors, this can be highlighted in the work of Kotelnikov et al. [106].

### 5.3. Multi-Modality in Medical Domain

Multi-modality refers to integrating data from multiple sources to improve the learning capabilities of the model. In the context of the medical domain, multi-modality allows one to point out the strengths of different imaging modalities. However, some challenges are still encountered, as discussed by Zhou et al. [107], Tawfik et al. [108], Li et al. [109], and Fawzi et al. [110]. The data heterogeneity of each modality (e.g., MRI, CT) provides unique and complementary information with distinct statistical probability distributions, making it challenging to integrate and synchronize data across modalities effectively. Algorithmic complexity and computational resources, particularly those that require real-time processing, add significant computational costs, which can be a barrier with limited resources. Another challenge is the scalability and model efficiency. Ensuring that multi-modal models can scale efficiently with increasing data volume and variety without loss in performance is a critical factor for their adoption. Robustness and generalization are other challenges that multi-modals face as there is a lack of generalization on the models and a gap between model training environments and real-world medical settings. Missing modalities can also often occur in many real-world scenarios, where some expected data modalities may be unavailable, which can degrade the model performance if not handled correctly. These challenges reveal the need for continuous research in data processing techniques, model architectures, computational strategies, and regulatory compliance to fully leverage the potential of multi-modal deep learning in the medical domain. Addressing these challenges will enhance the practical applicability of the models, ensuring that multi-modal DL can offer a valuable tool in clinical applications.

### 5.4. Tabular to Image Data in Medical Domain

Transforming tabular medical data into image formats presents unique challenges that must be addressed to fully harness the potential of image-based deep learning techniques. One major challenge is the design of effective encoding schemes that accurately represent complex medical data as images without losing critical information. Data dimensionality and sparsity also pose significant problems. Tabular medical datasets are often composed of hundreds of features (thus increasing the colinearity), each representing different aspects of patient health. Being able to efficiently compress tabular data into two-dimensional image formats without increasing the size of the model with irrelevant information or losing crucial information is a challenging task. Moreover, medical data can be highly sparse and imbalanced, which complicates the training of CNNs that perform best with dense, evenly distributed data. The interpretability of the transformations is another critical challenge. Medical decisions require high confidence and transparency to ensure trust and reliability. Transformations that obscure the meaning of the data could lead to diagnostics that are hard to interpret by healthcare professionals, potentially impeding their adoption. To embrace these challenges, the readers are directed to read the following works of Borisov et al. [111], Fonseca and Bacao [112], and Damri et al. [113].

### 5.5. Computational Efficiency

In the context of the medical domain, it is necessary that the algorithms are capable of executing in real time to ensure timely decision making and interventions. This requires not only fast data processing speeds but also algorithms that can efficiently handle large volumes of complex, multidimensional data generated by modern medical equipment. Therefore, a significant challenge lies in the development of scalable computational frameworks that maintain accuracy and speed despite the increase in data sizes and complexity. Future directions could include exploring the integration of quantum computing to manage these complexities more effectively and the use of distributed computing architectures to enhance the scalability and responsiveness of medical systems. So far, the state-of-the-art methods offer some strategies for efficient computation. Some of them are pruning [114], quantization [115,116], and knowledge distillation [117]. Additional works in the literature include the optimal search of an architecture model that enhances the best accuracy with the fastest inference time. These works can be found within the field of Neural Architecture Search (NAS). In the medical domain, NAS can be particularly beneficial for customizing neural network architectures to specific tasks such as medical image analysis, disease diagnosis, or personalized medicine. This field of work already presents relevant literature, as can be seen in the works of Zhu et al. [118] and Weng et al. [119]. However, there is a lack of research in the field of brain stroke, presenting an opportunity for future investigations.

### 5.6. Data Quality and Availability in Medical Domain

One of the foundations of effective ML and DL, particularly in the healthcare sector, is based on the quality and availability of data. High-quality data are crucial for training models that are accurate, reliable, and capable of generalizing from training environments to real-world applications. However, several challenges persist in ensuring the quality and accessibility of data, such as the following:

- Data Integrity and Accuracy: Ensuring data integrity involves maintaining the accuracy, consistency, and reliability of data throughout their lifecycle. In medical datasets, errors in data collection, processing, or handling can lead to significant biases in model training, potentially compromising patient safety. For example, in the work of Kruse et al. [120], the authors discuss several data challenges in healthcare, emphasizing the critical need for maintaining data integrity to avoid biases that could affect patient outcomes and safety.
- Data Annotation: Annotated medical data are crucial for supervised learning models. However, this process is very intensive and expensive, requiring expert knowledge that is occasionally lacking. For example the work of Rajpurkar et al. [121] highlighted the labor-intensive nature of preparing datasets for training diagnostic AI systems. Each image in the validation set had to be individually reviewed by multiple radiologists, a process that is both time-consuming and expensive due to the expertise required. As an alternative, to reduce the dependency on extensive annotated datasets, self-supervised and unsupervised learning techniques offer promising avenues. For a deeper understanding of self-supervised and unsupervised learning methods, the readers are directed to the following references: Doersch and Zisserman [122], Xie et al. [123].
- Data Diversity: Often, medical datasets may not adequately represent the diversity of the patient population, including variations in ethnicity, age, gender, and underlying health conditions. This lack of diversity can lead to models that perform well on the majority group but poorly on underrepresented groups. Chen et al. [124] address these concerns by investigating the sources of bias in datasets that lead to discriminatory outcomes in AI applications, emphasizing the importance of diverse data representation to prevent such biases. Moreover, the over-representation of one class further leads to a common problem of datasets exhibiting class imbalances. This imbalance can skew model training, leading to poor performance on less common but often more critical conditions.
- Data Accessibility and Sharing: While there are vast amounts of medical data, privacy concerns, regulatory restrictions, and proprietary interests often limit data sharing between institutions and researchers. Beam and Kohane [125] explore these issues by discussing the potential of big data in transforming healthcare and the barriers that inhibit its full utilization, including data sharing limitations.
- Legal and Ethical Considerations: The use of AI in healthcare raises significant ethical and legal challenges, particularly concerning data privacy, consent, and the potential for the misuse of sensitive health information. Cohen et al. [126] discuss the ethical and legal challenges associated with deploying AI in healthcare, highlighting the need for robust frameworks to manage these issues.

## 6. Answering the Research Questions

As a final discussion of this review, the following section focuses on answering the research questions formulated in Section 3.

### 6.1. RQ1—What Are the Cases Where Machine Learning and Deep Learning Are More Appropriate for Building a Robust and Accurate Model for Classification Problems?

After a careful review of the review papers concerning classification problems in brain stroke diagnosis, it is important to highlight that the efficiency of the computational models largely depends on the nature of the dataset used. Traditional machine learning algorithms such as DTs, RFs, SVMs, and GB are generally more effective with datasets composed of tabular data. These algorithms are highly effective at managing structured data, enabling them to effectively capture and model the intricate and often non-linear relationships among various variables that are typical in medical datasets. On the other hand, DL approaches, particularly CNNs, show greater suitability for image-based datasets. These models leverage their architecture, which is vital for interpreting the high-dimensional data typical of medical images, such as CT scans or MRIs. This ability is crucial for identifying subtle patterns indicative of strokes and classifying different types of strokes with high accuracy. Moreover, it is important to note here that traditional ML techniques might not capture complex patterns in high-dimensional spaces adequately, where DL models can autonomously learn and make predictions based on the hierarchical features of the data. While ML models often provide more interpretability than DL models, which is a significant advantage in clinical settings, as understanding the decision-making process is paramount for trust and ethical considerations, DL models typically offer superior performance on tasks involving complex visual data, sacrificing transparency for higher accuracy. Given the answer based on the comprehensive research conducted here, it is noteworthy to mention that the combination of both worlds, i.e., the integration of both tabular and imaging data to create hybrid models that utilize the strengths of both ML and DL approaches to therefore improve the robustness and accuracy of predictions by providing a more comprehensive view on diagnosing a given patient.

### 6.2. RQ2—What Are the Challenges and Limitations of Current AI Segmentation Techniques in Analyzing Complex Brain Imaging Data?

According to the review conducted, the integration of AI segmentation techniques into medical imaging analysis has significantly enhanced the capabilities of MRI and CT scans in lesion detection, crucial for the diagnosis and treatment planning of stroke. However, some challenges and limitations persist in the segmentation of complex brain imaging data. Concerning data complexity and quality, DL models require large volumes of high-quality, annotated imaging data to train effectively. However, the availability of such data is often limited due to privacy concerns, a lack of expertise in these annotations, and high costs of data curation. Moreover, for example, MRI scans, which provide a great soft tissue contrast, making them ideal for early stroke detection, can vary significantly in quality and detail. These adversities can affect the performances of DL models. Another well-known challenge pointed out in the literature is model generalization. For example, U-Net architectures, are prone to overfitting due to their complexity and depth of features. While these models perform well when the probability distribution is known, they tend to fail to generalize across datasets that differ slightly in terms of acquisition parameters or demographic characteristics of the subjects. This limitation is critical in clinical settings, in which the deployment of such models relies on how well they can generalize to other cases. Computational demands are also quite a challenge in DL since the computational requirements are significant. They require substantial GPU resources for processing, which can be a barrier in many cases when there is limited technological infrastructure. These limitations are discussed with the open challenges, where solutions such as the pruning or quantization of the models could be of valuable interest for limited hardware capabilities. Lastly, issues related to interpretability, also discussed previously on the open challenges, are a major concern for this kind of DL models, as most of them are of a black-box nature. The lack of interpretability can be a significant barrier in the decision-making process in clinical practice, where understanding the decision of the model is of paramount importance.

### 6.3. RQ3—Why Object Detection Studies Are Less Studied When Compared to Segmentation?

Based on this review, it was concluded that object detection and segmentation are both critical in medical imaging for enhancing diagnostic accuracy. However, several distinct factors have been identified that explain why segmentation studies are more prevalent than object detection. First was the clinical utility. Segmentation provides detailed maps of lesions that are critical for treatment planning, whereas object detection primarily identifies and locates lesions without detailing their extent. Secondly, in terms of data availability, high-quality annotation datasets are more common for segmentation tasks than object detection. Thirdly, in terms of feature complexity, stroke lesions display complex, variable features that are challenging to delineate using object detection. Moreover, segmentation approaches are better fit to be included in clinical workflows, offering valuable insights for immediate clinical intervention, unlike object detection, which lacks the granularity needed for clinical intervention.

### 6.4. RQ4—What Are the Most Prominent Challenges and Future Directions in Machine Learning and Deep Learning Considering Stroke Diagnosis?

To conclude this review, it is pertinent to address this RQ that highlights the significant challenges and illuminates the path forward in the application of ML and DL technologies in stroke diagnosis. This question not only captures the essence of our findings, based on what we could conclude from the review papers, but also sets the stage for addressing crucial obstacles and opportunities in future research. DL models in stroke diagnosis face challenges due to their black-box nature, impeding clinical trust. The effectiveness of these models depends heavily on the availability of high-quality, well-annotated data, which, in the real application of object detection, is more scarce compared with segmentation. Generative models such as GANs, VAEs, and diffusion models help with the augmentation of the datasets but can struggle with domain adaptation and generalization. Additionally, integrating multi-modal data and meeting the computation demands for real-time processing are significant challenges. Future advancements could include the development of transparent, explainable models and more efficient computation strategies such as quantum computing. Techniques for transforming tabular data into images without losing relevant information and NAS for optimizing the model designs are also promising directions. Addressing these issues could significantly enhance the accuracy, efficiency, reliability, and fairness of AI in stroke diagnosis.

## 7. Conclusions

We provided a comprehensive review by analyzing review papers on brain stroke analysis, focusing on classification, segmentation, and object detection. Through this review, we identified and analyzed 25 pertinent review papers. Most papers were selected based on a keyword search, citation count, and the FCWI. Each study was rigorously analyzed to gather extensive data and organized into comprehensive tables highlighting findings, strengths, limitations, and ongoing challenges.

This structured analysis enhanced our understanding of the current landscape and allowed us to infer that it was possible to answer the research questions posed at the beginning of this study. In the areas of classification and segmentation, our review highlights advancements in algorithmic precision and the integration of deep learning techniques that have significantly improved diagnostic accuracies. For object detection, although less studied, we identified significant potential for future applications that could substantially improve rapid diagnosis processes.

From our analysis of the review papers, it was possible to infer, and therefore discuss, pertinent open challenges and prospective directions for ML and DL in medical imaging, emphasizing the need for more transparent, explainable models to promote greater trust and integration into clinical practices. The exploration of advanced computational methods and innovative model architectures promises to address these issues effectively. Furthermore, based on our analysis of the review papers, we answered our proposed research questions, providing insights into the appropriate applications of ML and DL for classification, the challenges of current segmentation techniques, the reasons for the limited focus on object detection studies, and the prominent challenges and future directions in the field. Specifically, we highlighted the suitability of traditional ML for structured data and DL for image-based data, the need for high-quality annotated data for effective segmentation, the clinical utility driving the prevalence of segmentation over object detection, and the importance of developing explainable models and efficient computational strategies to enhance AI-driven stroke diagnosis.

To conclude this review, it is important to acknowledge our limitations. Firstly, our study was confined to papers published in English, potentially excluding significant research conducted in other languages and published in non-English journals. Secondly, our keyword search might have excluded relevant works that could enrich our comprehensive review. Thirdly, the papers reviewed were limited to those accessible through specific academic databases, potentially omitting relevant studies published in less accessible or non-indexed journals. Additionally, while our review provides a comprehensive examination of the literature from 2020 to 2024, it is important to acknowledge the inherent limitations associated with our methodology and scope. Our study is confined to English-language papers accessible through major academic databases, which may exclude significant research published in other languages or in less accessible journals. Additionally, the nature of academic publishing and our review’s cut-off date inherently limit our coverage of the latest developments that may emerge post publication. Recognizing these limitations is crucial for readers to understand the context and constraints under which this review was conducted. This acknowledgment not only highlights the potential gaps in our review but also underlines the dynamic and evolving nature of research in brain stroke diagnosis. 

## Figures and Tables

**Table 1:** Overview of the most common models, metrics, and losses used in classification, segmentation, and object detection problems.

	Classification		Segmentation	Object Detection
	**ML** ^1^	**DL**		
**Models**	LR DT RF SVM NB GB ANN ^1^	ANN ^2^ AlexNet [22] VGG [23] ResNet [24] Inception [25] DenseNet [26] EfficientNet [27]	FCN [28] U-Net [29] SegNet [30] DeepLab [31] Mask R-CNN [32]	Faster R-CNN [33] YOLO [34] SSD [35] RetinaNet [36] DETR [37]
**Metrics**	Accuracy Precision Recall F1 Score ROC-AUC Confusion Matrix		Pixel Accuracy Intersection over Union Dice Coefficient Boundary Accuracy	Precision and Recall Average Precision Mean Average Precision (mAP) Intersection over Union (IoU)
**Losses**	Binary Cross-Entropy Loss Hinge Loss Multi-Class Cross-Entropy Loss Categorical Cross-Entropy Loss Sparse Categorical Cross-Entropy Loss		Dice Loss Cross-Entropy Loss Jaccard Loss	Focal Loss Smooth L1 Loss Combined Objectness and Class Specificity Loss Region Proposal Network Loss

^1^ All algorithms of ML can be consulted in [12,13,14,15,16,17,18,19,20,21]. ^2^ ANN percepton based on ML and ANN hidden layer for DL.

**Table 2:** Summary of the key points of each review paper for classification.

Author, Year	N. Papers	Range	Paper Objectives
Sirsat et al. [1], 2020	39 papers	2007–2019	They conducted a systematic categorization of the studies into stroke prevention, diagnosis, treatment, and prognostication.
Wang et al. [39], 2020	18 papers	1990–2019	They provide a table summarizing the included studies, offering insights ML algorithm adopted, type of data, and predicted outcomes.
Gagana and Padma [40], 2021	10 papers	N/A	The primary goal was to identify and analyze ML techniques that have been proven effective in predicting strokes. The paper reviews past research focusing on mortality rates and functional outcomes.
Cui et al. [43], 2022	21 papers	2017–2022	They present a summary of five clinical applications of DL in acute ischemic stroke: early stroke diagnosis, automated ASPECT calculation, the detection of Large vessel occlusion, the evaluation of ischemic core and penumbra/prognosis, and the prediction of imaging function outcomes.
Ruksakulpiwat et al. [47], 2023	12 papers	2015–2021	Twelve studies were included, utilizing 15 different algorithms. The studies used a variety of input features, with age and gender being the most common.
Mushtaq and Saini [41], 2023	22 papers	N/A	The review aimed to analyze the different studies using the Healthcare Kaggle stroke dataset with various performance metrics.
Bashir et al. [42], 2023	12 papers	2019–2022	The paper reviews 12 studies on machine learning for stroke prediction, focusing on techniques, datasets, models, performance, and limitations.
Tan et al. [44], 2023	25 papers	2016–2022	They review several papers aiming to answer three research questions: **RQ1:** What are the data needed for predicting ischemic stroke using deep learning? **RQ2:** Which methods of deep learning have the best performance in terms of the accuracy of detecting ischemic stroke? **RQ3:** What is the prediction of ischemic stroke used for?
Bajaj et al. [45], 2023	10 papers	N/A	The authors divide the studies into ML and DL, discussing the advantages and disadvantages of these methods; they also present eight public datasets for stroke.
Daidone et al. [46], 2024	10 papers	2014–2020	The paper highlights the increasing adoption of ML techniques in stroke medicine, which facilitates the efficient analysis of large datasets and support advanced personalized and precision medicine approaches.

**Table 3:** Summary of the strengths and limitations of each review paper for classification.

Author, Year	Strengths and Limitations
Sirsat et al. [1], 2020	The paper categorizes four types of stroke that help the readers to dive into stroke management areas where ML has been applied It could further explore and discuss future research directions in more detail.
Wang et al. [39], 2020	First systematic review that reviewed not only the reporting quality of the ML studies but also the development of the ML models Including information on performance metrics would improve the understanding of the effectiveness of the models and clinical applicability
Gagana and Padma [40], 2021	The comparison includes metrics such as sensitivity, specificity, accuracy, and area under the ROC curve, which are crucial for evaluating model performance. The paper concludes without recommending a specific ML technique as superior for stroke prediction, suggesting that the choice depends on the scenario, datasets, and parameters.
Cui et al. [43], 2022	The paper highlights several key clinical applications of DL in stroke care, including early stroke diagnosis, automated ASPECTS calculation, and large vessel occlusion detection. While the review offers an overview of various DL models, the discussion might lack the depth required for readers seeking to understand its parameterization.
Ruksakulpiwat et al. [47], 2023	By reviewing studies that used various machine learning algorithms for different aspects of stroke classification, the paper provides a holistic view of the field’s current state. While acknowledging the need for larger datasets, the review could provide a more detailed discussion on the impact of data size and quality on ML model performance, which is crucial for real-world applications
Mushtaq and Saini [41], 2023	The paper clearly outlines several research gaps, such as the challenges of imbalanced datasets, the need to consider a wider range of medical attributes, and the importance of evaluating models based on execution time. Further research is necessary to determine the optimal combination of predictors and how best to integrate these predictors into clinical practice.
Bashir et al. [42], 2023	The paper identifies crucial research gaps and challenges, such as the lack of reliable biomarkers, limited sensitivity and availability of imaging techniques, and the need for standardized diagnostic criteria. While accuracy is frequently mentioned, there is limited discussion on the importance of other performance metrics such as precision, recall, F1 score, and their relevance in the clinical diagnosis of stroke.
Tan et al. [44], 2023	Offers an extensive analysis of the types of data (e.g., CT scans and MRI images) used in the literature for predicting ischemic stroke, highlighting the prevalence and significance of each type. The paper focuses on the advantages and application of deep learning techniques but does not provide a detailed exploration of the limitations, challenges, and potential pitfalls of applying these methods in clinical settings.
Bajaj et al. [45], 2023	Offers detailed tabular forms comparing the performances of ML and DL models. Moreover, it includes a valuable list of open datasets. The review does not address challenges for future works on either ML or DL models for stroke detection.
Daidone et al. [46], 2024	The paper thoroughly examines the range of machine learning techniques from logistic regression to advanced neural networks, providing an overview of their applications in stroke medicine. While the paper presents a table with principal fields of application of ML in stroke medicine, it does not present the metrics adopted or results for further discussion.

**Table 4:** Summary of the challenges/opportunities of each review paper for classification.

Author, Year	Challenges/Opportunities
Sirsat et al. [1], 2020	Challenges are not mentioned by the authors
Wang et al. [39], 2020	Challenges are not mentioned by the authors
Gagana and Padma [40], 2021	While ML models exhibit promising results in stroke prediction, challenges such as data standardization, model validation, and the need for real-time applicability persists.
Cui et al. [43], 2022	Lack of interpretability still observed in DL models. Confidential issues that prevent the external validation of the models and therefore poor generalization Lack of interpretability still observed in DL models Need for larger datasets to validate efficiency of DL models as well as explore different scenarios.
Ruksakulpiwat et al. [47], 2023	Choosing the most appropriate ML algorithm for a specific dataset and objective is complex due to the diverse nature of stroke-related data.
Mushtaq and Saini [41], 2023	Improved on the issue of imbalanced datasets, which is used in previous works and included other ranges of features (e.g sytolic and diastolic blood pressure, and pulse) that could enhance the model performance. The execution time of the model, an important aspect for real-time applications.
Bashir et al. [42], 2023	There is a notable absence in the literature of reliable biomarkers for the early detection of brain stroke, hindering prompt and accurate diagnosis. Current imaging techniques suffer from limited sensitivity, making it challenging to detect stroke early. The accessibility of advanced imaging techniques is limited, especially in resource-constrained settings. There is no universally accepted set of diagnostic criteria for stroke, complicating the consistency of diagnosis across different healthcare settings. The biological and physiological mechanisms underlying stroke are not fully understood, limiting the development of targeted interventions.
Tan et al. [44], 2023	Challenges are not mentioned by the authors
Bajaj et al. [45], 2023	Challenges are not mentioned by the authors
Daidone et al. [46], 2024	The lack of standardized data formats and interoperability across systems poses significant challenges in developing and applying effective ML models in stroke medicine as well as model validation and generalization

**Table 5:** Summary of the key points of each review paper in the field of segmentation.

Author, Year	N. Papers	Range	Paper Objectives
Karthik et al. [51], 2020	113 papers	Until 2020	They evaluate the advancements of DL models in the detection and segmentation of brain stroke lesions by exploring different architectures with focus on CNNs and FCNs, applied across modalities such as CT and MRI.
Isa et al. [49], 2020	23 papers	N/A	The paper reviews both automated and semi-automated segmentation methods, discussing the advantages and limitations of existing algorithms, and also presents a comparative review of the studies in terms of performance modeling.
Liu et al. [53], 2020	more than 100 papers	N/A	They provide a comprehensive review of U-Shaped networks used in medical imaging segmentation.
Inamdar et al. [52], 2021	177 papers	2010–2021	The authors aimed to review advancements in computer-aid diagnosis for acute brain stroke, underlining the modalities and methodologies used in neuroimaging for stroke identification and classification.
Saad et al. [48], 2021	13 papers	2015–2021	The review paper aims to evaluate existing image segmentation techniques applied to MRI scans in diagnosing brain stroke lesions. The paper discusses manual, semi-automatic, and fully automatic segmentation techniques.
Thiyagarajan and Murugan [58], 2021	4 papers	N/A	The paper systematically evaluates the different techniques employed in the segmentation and classification of ischemic stroke lesions using MRI technology. It discusses automated and semi-automated techniques that improve the accuracy and efficiency of diagnosing ischemic stroke.
Subudhi et al. [50], 2022	153 papers	1990–2021	The paper synthesizes the current knowledge on the application of ML techniques to MRI-based ischemic stroke characterization. The paper also discusses various ML algorithms used in the segmentation and classification of ischemic stroke lesions.
Jyothi and Singh [56], 2023	60 papers	2014–2021	The main objective is to evaluate and review the existing techniques for MRI-based brain tumor segmentation, which includes traditional automated methods as well as modern DL models.
Ahmed et al. [57], 2023	34 papers	2019–2022	The main goal is to evaluate the efficiency and effectiveness of AI-based segmentation tools developed and tested using the ATLAS dataset.
Abbasi et al. [54], 2023	22 papers	2017–2023	The primary aim of the review is to evaluate the performance of various DL models in segmenting ischemic stroke lesions from brain MRI and CT images. The paper covers significant studies that use DL for stroke lesion segmentation, providing a critical analysis of methodologies, datasets, and results.
Malik et al. [55], 2024	28 papers	2018–2023	The main objective of the paper is to present a comprehensive survey of DL applications in stroke lesion segmentation using MRI and CT images. The paper covers several DL models such as CNNs and transformers used in stroke lesion segmentation.

**Table 6:** Summary of the strengths and limitations of each review paper on segmentation.

Author, Year	Strengths and Limitations
Karthik et al. [51], 2020	It successfully breaks down complex methodologies and compares them across different criteria, providing a solid foundation for both new and established researchers. The authors claim that the keyword usage might have limited them to a certain type of studies, hence the neglect of relevant works.
Isa et al. [49], 2020	Thoroughly covers a wide range of segmentation techniques and evaluates them critically. Image acquisition and pre-processing methods could be deeply investigated such as in terms of how they would be relevant to the performance of the models.
Liu et al. [53], 2020	Very comprehensive and complete review of U-shape networks and their variations. Extensive perspective of U-shape applications in different areas. The discussion on the problems involved in the complexity of the image segmentation tasks to be used in clinical diagnosis could be a more detailed analysis.
Inamdar et al. [52], 2021	In-depth coverage of imaging modalities and diagnostic techniques. Comprehensive review tables summarize the contributions, providing clarity on the performance and reliability of different methods. While the authors provide a very insightful in-depth discussion of the reviewed papers, the authors could provide a more thorough discussion of open challenges and future directions that could be provided, mainly on the heterogeneity of the data from segmentation images.
Saad et al. [48], 2021	The paper covers a wide range of segmentation techniques, providing a thorough dive into each method and use case. A lack of a well-defined search strategy, which may lead the readers to struggle to understand the most relevant papers selected for the research.
Thiyagarajan and Murugan [58], 2021	Thoroughly assesses both established and emerging techniques, providing a detailed comparison of their clinical utility. The selection of only a few studies. Although the revision is thorough and accurate, a lack of a broader revision of other papers covering the same fields is missing.
Subudhi et al. [50], 2022	Detailed discussion on the advantages and limitations of various machine learning techniques as well as segmentation techniques. More detailed discussions on future directions as well as open challenges are missing.
Jyothi and Singh [56], 2023	The paper provides a broad revision of segmentation technologies, giving a detailed comparison of their performance. The thorough discussion of conclusions regarding unsupervised methods to generate labels to assist segmentation tasks could be better discussed and some challenges could be provided for future research.
Ahmed et al. [57], 2023	The paper provides a broad variety of studies, providing robust comparisons of different methodologies. The authors acknowledge some limitations such as the consideration of only one dataset (ATLAS), and lack of discussion on interpretable models (explainable AI).
Abbasi et al. [54], 2023	The paper provides a critical evaluation of MRI and CT modalities, which yields insightful guidelines for medical practitioners and researchers. Limitations and future directions on ischemic stroke segmentation are slightly discussed and could be further detailed.
Malik et al. [55], 2024	The paper provides a comprehensive review of DL techniques in the context of stroke lesion segmentation. The effective synthesis of comparative data between MRI and CT modalities, offering valuable insights for medical practitioners and researchers. The paper presents some limitations such as the inclusion of only English technical studies, which offers a partial overview of the topic, the limited keyword search, and the inclusion of studies solely on lesion segmentation.

**Table 7:** Summary of the challenges/opportunities of each review paper on segmentation.

Author, Year	Challenges/Opportunities
Karthik et al. [51], 2020	The authors claim the lack of public datasets as a challenge as well as the high class imbalance among them. Transfer learning can be problematic since a great part of the datasets are trained on ImageNet. Many DL approaches are reported on for the detection, classification, and segmentation of ischemic and hemorrhagic stroke, but there exists a vital need to develop DL regression algorithms in the assessment of modified Rankin scale to help physicians decide on appropriate treatment procedures for recovery patients.
Isa et al. [49], 2020	Medical image fusion to reduce the time to diagnose multiple modalities. The usage of the color segmentation model to improve the performance of the model on the identification of the damaged zones.
Liu et al. [53], 2020	The paper identifies ongoing challenges such as handling limited training samples, improving model generalization, and reducing computational costs for 3D image segmentation.
Inamdar et al. [52], 2021	The authors denote challenges in the application of ML/DL techniques to stroke diagnosis, such as data heterogeneity, model interpretability, and computational demands.
Saad et al. [48], 2021	The authors do not present any challenges
Thiyagarajan and Murugan [58], 2021	The authors emphasize the intrinsic challenges in segmenting stroke lesions, such as the variability in lesion appearance across patients and the subtle differences between lesion tissues and normal brain tissues. The development of more robust models that can handle the heterogeneity of stroke lesions and the integration of multimodal imaging data. Model generalization to perform well across diverse datasets.
Subudhi et al. [50], 2022	The authors conclude with future directions emphasizing the need for efficient and robust deep learning models for quantitative brain MRI analysis.
Jyothi and Singh [56], 2023	The authors conclude with a discussion on the current challenges in brain tumor segmentation, which include data scarcity, the variability of MRI scans, and the need for models that can generalize across different imaging protocols. The authors also suggest future directions, the development of more robust and efficient DL models, the improvement of dataset quality, and exploring novel network architectures and training strategies.
Ahmed et al. [57], 2023	The authors highlight the need for more robust AI architectures capable of handling the spatial heterogeneity of chronic stroke lesions. The authors also mention that future research could focus on developing new models, improving dataset quality, and exploring the integration of multimodal imaging data.
Abbasi et al. [54], 2023	The authors addresses challenges such as data scarcity, class imbalance, and the generalizability of models across different datasets and imaging modalities.
Malik et al. [55], 2024	The authors claim a need to analyze advanced pre-processing techniques in conjunction with lesion segmentation as well as the need for the improvement of transfer learning for model generalization.

**Table 8:** Summary of the key points of each paper in the field of object detection.

Author, Year	N. Papers	Range	Paper Objectives
Jiang et al. [59], 2023	N/A ^1^	N/A	They aimed to provide an in-depth review of deep learning-based methods for multiple-lesion recognition from medical images. This includes classification, detection, and segmentation techniques.
Sailaja and Pattani [60], 2023	N/A	N/A	The primary objective was to develop a deep learning model incorporating YOLOv5 and SSD for predicting brain strokes from MRI images.
Ayoub et al. [61], 2023	N/A	N/A	The primary objective was to enhance the ViT architecture for the multi-slice classification and localization of brain strokes using CT scans.
Zhang et al. [62], 2021	N/A	N/A	The primary objective was to develop automated lesion detection methods for ischemic stroke using deep learning techniques, specifically applying Faster R-CNN, YOLOv3, and SSD networks.

^1^ Although the number of papers they analyze are not mentioned, the authors retrieved papers related to the recognition method based on deep learning with human main organs and tissues in the last two years.

**Table 9:** Summary of the strengths and limitations of the papers on object detection.

Author, Year	Strengths and Limitations
Jiang et al. [59], 2023	The paper covers a wide range of deep learning techniques and their applications in multiple-lesion recognition. The analysis includes various body regions and types of lesions, providing a thorough understanding of the field.
Sailaja and Pattani [60], 2023	The model demonstrates high accuracy (96.43%) in predicting brain strokes, indicating its effectiveness in identifying stroke lesions. The study is based on a sample dataset of 459 MRI stroke images, which may not be sufficient to generalize the findings across diverse populations and imaging conditions.
Ayoub et al. [61], 2023	The proposed framework achieved an overall accuracy of 87.51% in classifying brain CT scan slices, demonstrating its effectiveness in stroke diagnosis. The study was based on a dataset of 730 patients, which, while diverse, may not fully represent the wide range of variations in patient demographics and imaging conditions.
Zhang et al. [62], 2021	The SSD network achieved the highest precision of 89.77% for lesion detection, demonstrating the effectiveness of the approach. Although substantial, the dataset is still relatively limited in diversity, and further validation on larger and more diverse datasets is necessary.

**Table 10:** Summary of the challenges/opportunities of each paper on object detection.

Author, Year	Challenges/Opportunities
Jiang et al. [59], 2023	Ensuring a diverse and high-quality dataset is crucial for training robust models. The variation in imaging modalities, patient demographics, and disease presentations adds to the challenge. Developing more sophisticated data augmentation techniques and generating synthetic data can help mitigate the issues caused by limited datasets. Utilizing pre-trained models and transfer learning can improve model performance, especially when working with small datasets.
Sailaja and Pattani [60], 2023	Adapting and integrating the model into existing clinical workflows and ensuring that it meets the regulatory and ethical standards are significant challenges. Conducting real-world clinical trials and validations can enhance the credibility and adoption of the model in healthcare settings.
Ayoub et al. [61], 2023	Ensuring that the model generalizes well across different populations and clinical settings remains a significant challenge. Future research can focus on validating the model on larger and more diverse datasets to further assess its robustness and generalizability.
Zhang et al. [62], 2021	Adapting the deep learning models for seamless integration into clinical workflows, including handling variations in MRI machines and protocols, is crucial for practical implementation. Enhancing the interpretability and explainability of the AI models will help gain trust from healthcare professionals and improve adoption rates.

## Data Availability

No new data were created. All data mentioned herein are referenced with the appropriate paper and/or website link.

## Abbreviations

ML	Machine Learning
MRI	Magnetic resonance imaging
CT	Computed Tomography
DL	Deep Learning
AI	Artificial Intelligence
xAI	Explainable AI
SVM	Support Vector Machine
DT	Decision Tree
RF	Random Forest
NB	Naïve Bayes
GB	Gradient Bosting
LR	Logistic Regression
CNN	Convolution Neural Network
GAN	Generative Adversarial Network
VAE	Variational Autoencoder
ANN	Artificial Neural Network
NAS	Neural Architecture Search
RQ	Research Question
ViT	Vision Transformer
NIHSS	National Institutes of Health Stroke Scale
CPSS	China National Stroke Registry
BraTS2020	Brain Tumor Segmentation
ATLAS	Anatomical Tracings of Lesions After Stroke
HRS	Health and Retirement Study
mAP	Mean Average Precision
IoU	Intersection over Union
FCWI	Frequency of Cited Works Indicator
YOLO	You Only Look Once
SSD	Single-Shot Detector
Faster R-CNN	Region Convultional Neural Networks
